# Creatine-precursor exposure in ewe twin pregnancy directs metabolic effort and reworks the maternal–fetal vascular system, involving genetic markers that modulate the creatine pathway

**DOI:** 10.3389/fvets.2026.1795381

**Published:** 2026-07-09

**Authors:** Bruna Vitória de Freitas Alves, Camila Muniz Cavalcanti, Alfredo José Herrera Conde, Larissa Fernandes Baia Cesar, Marta da Costa Sousa, Jhennyfe Nobre de Sena, Yohana Huicho Miguel, Fernando Felipe da Silva Pereira, Louhanna Pinheiro Rodrigues Teixeira, Lucy Vanessa Sulca Ñaupas, Ana Flávia Bezerra da Silva, Ana Paula Ribeiro Rodrigues, César Carneiro Linhares Fernandes, Juliana Paula Martins Alves, Dárcio Ítalo Alves Teixeira, Davide Rondina

**Affiliations:** 1 School of Veterinary Medicine, Ceará State University (UECE), Fortaleza, Ceará, Brazil; 2 Experimental Biology Center, University of Fortaleza, Fortaleza, Ceará, Brazil; 3 Centre of Health Science, University of Fortaleza, Fortaleza, Ceará, Brazil

**Keywords:** angiogenesis, creatine, fetal development, sheep, twin pregnancy

## Abstract

**Introduction:**

Although creatine has a recognized role during gestation, there is still little information about multiple pregnancies. Especially in small ruminants, these pregnancies increase nutritional needs and the incidence of pre- and postpartum metabolic or reproductive pathologies. Thus, the main objective of this study was to address this gap by providing information on the impact of dietary supplementation with the creatine precursor guanidinoacetic acid (GAA) on the maternal–fetal response during late gestation in twin-bearing ewes.

**Methods:**

A total of 15 twin-bearing ewes were allocated to a baseline total mixed ration (TMR) diet group (BSTMR, *n* = 8) or a group whose TMR was supplemented daily with 0.9 g/kg of a diabetes mellitus (DM) diet of guanidinoacetic acid TMR (GATMR, *n* = 7) from 100 days of gestation until lambing.

**Results:**

The GATMR ewes adapted their diet selectivity and exhibited a smaller reduction in loin and kidney fat thickness. GAA supply increased peripheral levels of albumin, globulin, glucose, and cholesterol. In the GATMR group, a reduced development of the uterine artery, umbilical vascular system, caruncle thickness, and fetal heart rate was observed. Cotyledonary capillary structure in GATMR showed slower growth and sub-expression of *FGF2* and *FLT1*, whereas the antiangiogenic factor *ODC1* had a higher expression. The GATMR group also exhibited lower expression of the creatine regulator GAMT and a higher expression of the creatine kinase *CKM* gene.

**Discussion:**

Thus, the evidence indicates that GAA is effective in twin pregnancies even when provided in a rumen-unprotected form. The results also highlight the need for further studies to better modulate the dosage, adapting it to the demands of pregnancy.

## 1 Introduction

In multiple pregnancies, nutritional requirements and the incidence of metabolic and reproductive disorders increase during the pre- and postpartum periods. In late gestation, nutritional imbalances can lead to significant economic losses in sheep production (1). In sheep, nutritional prediction models are based on factorial approaches that account for gestational age and estimated litter weight; however, they have limited ability to prevent metabolic disorders, particularly in multiple pregnancies (2, 3).

During this period, the placenta undergoes intense vascularization, regulated by angiogenic factors, hormones, and genes related to nutrition, to optimize efficiency and meet the increasing nutrient demand. Among the important signaling pathways for angiogenesis are the action of *vascular endothelial growth factor (VEGF),* which promotes angiogenesis by binding to its *receptor KDR*. In contrast, its other *receptor, FLT1*, acts as a negative regulator, and *fibroblast growth factor 2 (FGF2)*, which contributes to the initial stimulation of placental vascularization (4–6). Furthermore, the *IGF2-H19 gene complex* links placental nutrient supply with fetal nutrient demands for growth (7) and coordinates the molecular mechanisms by which environmental signals alter the placenta’s capacity for nutrient transport. The *IGF-2* gene regulates placental growth, and in rodents, its expression is associated with reduced placental size under conditions such as nutrient restriction and glucocorticoid administration (8, 9).

In this context, the importance of creatine during pregnancy is widely recognized, as it promotes and regulates energy production, thereby meeting the energy demands of the fetus (10). In humans, the metabolic demands of the placenta rely on the creatine kinase circuit to sustain placental bioenergetics, and a hypoxic placenta may exhibit greater dependence on creatine and this circuit to maintain ATP homeostasis (11, 12). In swine, an efficient strategy for providing creatine during pregnancy is dietary supplementation with its precursor guanidinoacetic acid (GAA) (13). In ruminants, this strategy has been explored in recent years, based on evidence of partial degradation at the ruminal level (47 and 49% ruminal escape) (14), thereby enabling the escape of GAA and its availability for metabolic conversion to creatine via the *creatine transporter SLC6A8* (15). GAA is synthesized endogenously from arginine and glycine through the action of the enzyme *L-arginine: glycine amidinotransferase (AGAT)*, which transfers an amidino group from arginine to glycine. Following its production, GAA is methylated by *S-adenosyl-L-methionine: N-guanidinoacetate methyltransferase (GAMT)* to form *S-adenosylhomocysteine (SAH)* and creatine at the hepatic level. This mechanism is important to ensure that ATP reserves are available when needed (12, 16).

However, creatine can be harmful to both the fetus and the mother. To minimize the risk of severe negative energy balance, it is essential to adapt dietary energy levels to the increased needs of late pregnancy. The maternal system can interpret high energy availability during late pregnancy as a signal to accumulate reserves in the form of adipose tissue. At the fetal level, higher urinary excretion of GAA and lower urinary excretion of creatine have been reported in premature infants (17), possibly due to the newborn’s limited capacity to methylate GAA to form creatine, and elevated GAA levels may be neurotoxic (18). In ruminants, excessive prepartum body condition or marked fat and muscle mobilization at parturition predispose females to metabolic risks that impair reproductive performance. Changes in the lipid profile of pregnant women, besides contributing to metabolic disorders during gestation, also predict obesity in offspring during childhood (19).

The few studies available on the use of GAA or creatine during gestation do not focus on species characterized by multiple births or twin births, such as sheep. In this type of pregnancy, nutritional demands are amplified, as is the occurrence of prepartum and postpartum metabolic or reproductive pathologies (17, 20). Furthermore, there is still little information regarding the use of GAA in reproduction and even less during gestation. The main obstacle to the use of GAA and, consequently, creatine in animal metabolism is the timing of application and dosage. Previous studies in ovine used GAA dosages of 0.9 g/kg of dry matter diet for 60 days, with proven effects on nutrient digestibility and feed efficiency (18), which may pose a problem, given that reproductive processes have distinct physiological demands. In pregnant cows, Sousa et al. (19) used 0.2% GAA in the diet to evaluate metabolism and placental vascularization at the end of gestation, observing that supplementation favored the efficient use of arginine in nitric oxide (NO) synthesis and increased placental vascularization. Another issue concerns GAA’s ability to maintain its action over longer periods, such as throughout gestation, and the consequences this may entail. It is known that nutritional stimuli in animals with adequate energy status do not persist for extended periods but instead have a shorter duration of action.

Based on the scenario described above, we hypothesize that the administration of GAA in the diet, as a precursor of creatine, during the last third of gestation in twin-bearing ewes, is indeed effective in supporting maternal energy demand and efficiently sustaining fetal development. Thus, the present study aimed to examine the effect of including ruminal-unprotected GAA in the diet of twin-bearing ewes during the last third of gestation on the maternal metabolic profile and feeding behavior, as well as the impact of this product on maternal–fetal communication through ultrasonographic evaluation, immunofluorescence analysis of the fetal cotyledonary vascular structure, and gene expression of angiogenic markers and mediators of the fetal creatine pathway system.

## 2 Materials and methods

### 2.1 Facilities, animals, experimental conditions, and feeding management

The study was conducted at the experimental facilities of the School of Veterinary Medicine, State University of Ceará, Brazil. A total of 45 adult, multiparous Santa Inês ewes from the university flock were used in the experiment. All ewes were prepared for mating by synchronizing estrus and follicular waves using a hormonal protocol. An intravaginal sponge impregnated with 60 mg of medroxyprogesterone acetate (Progespon®, Zoetis, São Paulo, Brazil) was used. After 6 days, the sponge was removed, and 300 IU of equine chorionic gonadotropin (No-vormon®, Zoetis, São Paulo, Brazil) and 0.125 mg of PGF2α (Sincrocio®, Ourofino, São Paulo, Brazil) were administered. Subsequently, the ewes were mated with a proven-fertility Dorper ram. A total of 25 days after mating, pregnancy diagnosis was performed by ultrasonography, and only ewes with twin pregnancies (*n* = 15) were included in the study due to the greater metabolic challenge. The ewes confirmed as pregnant with twin lambs (*n* = 15) were then blocked by body weight into subgroups and kept in collective pens (two or three animals per pen) with concrete floors, receiving water and mineral salt ad libitum. All ewes received the same diet composed of a total mixed ration (TMR) based on corn silage and concentrate. From day 100 of gestation, the TMR was formulated to meet the nutritional requirements of adult pregnant sheep with twin lambs (2) for initial and late gestation. The particle size of the TMR was monitored using a Penn State particle separator, following the methodology described by Kononoff et al. (21). At 145 days of gestation, lambing was induced by intramuscular administration of 10 mL of dexamethasone (Azium® Solução; MSD, Brazil) and 1 mL of prostaglandin (Sincrocio®, Ourofino; Brazil). After birth, lambs were weighed.

### 2.2 Experimental design

**Figure 1:**
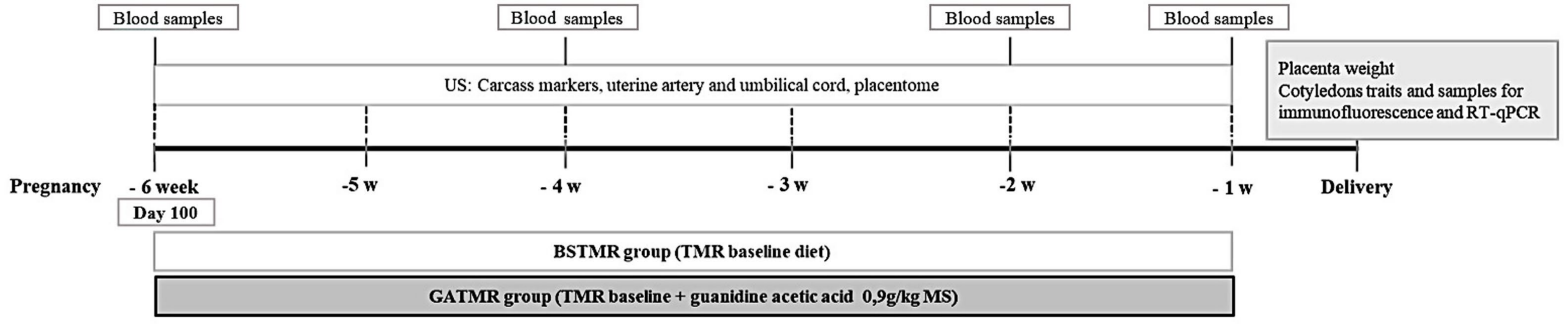
Timeline of experimental stages, including guanidinoacetic acid (GAA) supply.

On 100 days of gestation (6 weeks) (Figure 1), ewe subgroups were assigned to two nutritional treatments, homogeneous for weight (overall mean ± standard deviation [SD]: 51.7 ± 5.2 kg, *p* = 0.873). The control group (BSTMR, *n* = 8) received the baseline diet described above. The guanidinoacetic acid TMR (GATMR) group (*n* = 7) received the baseline diet supplemented daily with 0.9 g/kg of dietary diabetes mellitus (DM) of rumen-unprotected guanidinoacetic acid. Feed was offered twice daily, at 08:00 and 15:00, until lambing (Figure 1). All ewes were fed *ad libitum*, allowing up to 10% refusal (as fed), and intake was monitored daily throughout the experimental period. Guanidinoacetic acid in powder form (GuanAMINO®, Feed Grade 96.0%, Evonik Leading Beyond Chemistry, Hanau, Germany) was distributed in equal doses across each of the two daily meals.

### 2.3 Blood sampling, metabolite markers, and glutathione peroxidase assay

Blood samples were collected every 3 weeks (Figure 1). A 4 mL Vacutainer tube with lithium heparin anticoagulant (FIRSTLAB®, Disera Tıbbi Malzeme Lojistik San. Tic. A.Ş., Izmir, Türkiye) was used for collection. Blood was collected in the morning after fasting via jugular vein puncture and centrifuged at 3,000 rpm for 10 min to obtain plasma. Samples were stored in duplicate at −20 °C and subsequently analyzed for cholesterol, glucose, triglyceride, protein, albumin, urea, creatinine, glutamic-oxaloacetic acid transaminase (GOT), and glutamic-pyruvic acid transaminase (GPT). Analyses were performed by spectrophotometry on an automated biochemical analyzer (Mindray® BS 120, Mindray Biomedical Electronics Co., Shenzhen, China), using commercial kits (Bioclin®, Quibasa, Minas Gerais, Brazil). The kit had the following sensitivities and intra−/inter-assay coefficients of variation (CV): (1) Cholesterol, sensitivity of 1.472 mg/dL, intra-assay CV 1.35%, and inter-assay CV 1.85%. (2) Glucose, sensitivity of 1.31 mg/dL, intra-assay CV 2.59%, and inter-assay CV 0.78%. (3) Triglycerides, sensitivity of 2.58 mg/dL, intra-assay CV 0.59%, and inter-assay CV 0.54%. (4) Proteins, sensitivity of 0.043 g/dL, intra-assay CV 0.46%, and inter-assay CV 2.24%. (5) Albumin, sensitivity of 0.039 mg/dL, intra-assay CV 0.43%, and inter-assay CV 0.21%. (6) Urea, sensitivity of 1.514 mg/dL, intra-assay CV 2.96%, and inter-assay CV 1.17%. (7) Creatinine, sensitivity of 0.034 mg/dL, intra-assay CV 0.89%, and inter-assay CV 1.06%. (8) GOT, sensitivity of 1.756 U/L, intra-assay CV 0.73%, and inter-assay CV 0.99%. 9) GPT, sensitivity of 1.9543 U/L, intra-assay CV 2.83%, and inter-assay CV 0.44%. Globulin was calculated as the difference between total protein nd albumin. Glutathione peroxidase (GPx) was analyzed using a commercial kit (Randox Laboratories, Crumlin, UK), with a sensitivity of 75 U/L and intra- and inter-assay CV < 5%.

### 2.4 Carcass markers measurements

Every week until lambing (Figure 1), the depth of the lumbar region and perirenal fat thickness were measured as proposed by Morales-Martinez et al. (22) and Wang et al. (23), using B-mode ultrasound equipment with a 5 MHz linear probe (model Z5 Vet; Mindray Bio-Medical Electronics Co., Shenzhen, China). A convex transducer operating at 3.5 MHz (model Z5 Vet; Mindray Bio-Medical Electronics Co., Shenzhen, China) was used for kidney imaging. Images were captured in triplicate and measured using previously calibrated ImageJ software (version 1.5 g, ImageJ, National Institutes of Health, Millersville, United States). During evaluation, each ewe was kept stationary, the areas on the right side of the body were shaved, and gel was used as a coupling agent to improve image quality.

### 2.5 Uterine artery diameter and hemodynamic attributes

Uterine blood flow was measured weekly until lambing using pulsed color Doppler ultrasound (model Z5 Vet; Mindray Bio-Medical Electronics Co., Shenzhen, China) at 5.7 MHz and 28% color gain to visualize the uterine artery, measure its diameter, and capture pulsatile waves. Doppler velocimetric parameters were obtained from an average of three waves.

### 2.6 Placentome growth and caruncle thickness

Placentome size and caruncle thickness were measured weekly using B-mode ultrasonography (Figure 1). Images were acquired with an ultrasound machine (model Z5 Vet; Mindray Bio-Medical Electronics Co., Shenzhen, China), with the ewe in an upright position. The average diameter of the placentome was determined by randomly selecting three placentomes at each examination.

### 2.7 Umbilical vascular system development

Every seven days (Figure 1), the diameters of the umbilical vein and artery were measured using color Doppler ultrasonography with a 5 MHz convex transducer (model Z5 Vet; Mindray Bio-Medical Electronics Co., Shenzhen, China). Using D-mode, vessel diameters were obtained after freezing a cross-sectional image. The larger vascular structures were identified as arteries and the smaller as veins. For data analysis, the average of the two diameter measurements was calculated for each vessel.

### 2.8 Umbilical artery hemodynamics and fetal heart rate

Weekly, pulsatile waves from the largest umbilical artery were recorded in D-mode in an adequate portion of the cord, and hemodynamic parameters were determined. Scanner settings were calibrated as follows: angle 30°, sampling frequency 5.0 MHz, and color gain 32%. During data collection, the average of three separate cardiac cycles was calculated at each assessment. Fetal heart rate was measured using the same ultrasound equipment and at the same interval.

### 2.9 Placenta and cotyledon features measured at lambing

At lambing, the placenta was collected under sterile conditions and weighed (Figure 1). Cotyledons were classified into types A, B, C, and D according to their morphological characteristics (24), counted, and weighed by type. Then, five cotyledons of each type were randomly selected, weighed individually, and measured for length, width, and depth using a caliper.

### 2.10 Cotyledon capillary vascular structure by immunofluorescence analysis

Type C cotyledons were fixed in 4% paraformaldehyde (pH 7.2) for 4 h, dehydrated, cleared, paraffin-embedded, and sectioned to 5 μm thickness. Sections were mounted on positively charged slides and processed for Proliferating Cell Nuclear Antigen (PCNA) and Cluster of Differentiation 31 (CD31). Antigen retrieval was performed by incubating slides in 0.01 M sodium citrate buffer (pH 6) for 5 min in a pressure cooker. After cooling, slides were washed in Phosphate-Buffered Saline (PBS) and blocked for 1 h at room temperature using PBS containing 1% (w/v) Bovine Serum Albumin (BSA). After antigen retrieval, the slides were incubated overnight at 4 °C with primary antibodies against CD31 (blood vessel marker; 1:600; rabbit polyclonal; Abcam ab28364) and anti-PCNA (1:250; rabbit polyclonal; Abcam ab18197). Secondary Immunoglobulin G (IgG) antibody (1:200; Alexa Fluor® 488; Abcam ab150077) was applied for 2 h at room temperature, slides were washed in PBS, and mounted with Fluoroshield Mounting Medium with DAPI® (Abcam ab104139). Mouse spleen fragments (PCNA and CD31) served as positive controls, while sections processed without the primary antibody served as negative controls.

For analysis, images of 16,000 μm^2^ were captured at 40 × magnification using a fluorescence microscope (Eclipse E200, Nikon). CD31 labeling was used to evaluate the total number of capillaries per area (capillary number density [CND]) and total capillary circumference (capillary surface area density [CSD]), while PCNA labeling was used to count proliferating cells per area. Both were analyzed using ImageJ® software (version 1.54 g, National Institutes of Health, Millersville, PA, United States). Average capillary size was calculated as the average cross-sectional area per capillary (APC) by dividing CAD by CND, as proposed by Borowicz et al. (25).

### 2.11 RNA isolation and reverse transcription real-time quantitative polymerase chain reaction (RT-qPCR) of genetic markers in fetal cotyledonary tissue

The RNA was extracted from type C cotyledons using Trizol® reagent (Invitrogen, Carlsbad, CA, USA). RNA concentration was determined using a NanoDrop® 2000 spectrophotometer (Thermo Fisher Scientific, Waltham, MA, United States). cDNA synthesis was performed using 1 μg of total RNA with the High-Power Reverse Transcription Kit (Thermo Fisher Scientific®, Vilnius, Lithuania). The genes evaluated are listed in Table 1 and are associated with guanidinoacetic acid and creatine metabolism *(GAMT, GATM, CKM)*, angiogenic factors *(FGF2, FLT1, VEGFA, ODC1)*, creatine transport *(SLC6A8)*, and nutrients *(SLC2A1, SLC7A1)*. *RPS18* was used as an endogenous control because its expression remains unchanged in response to dietary supplementation. Primers were designed using Primer-BLAST (NCBI GenBank), specific to the species *Ovis aries*. Table 2 presents the phases and temperatures of the RT-qPCR cycles. The ∆∆CT method was used to transform cycle threshold data into normalized messenger RNA (mRNA) expression levels.

**Table 1:** Forward and reverse ovine primer sequences, gene bank, and references of genes used in RT-qPCR.

Gene	Length	Direction	Primer (5’to 3′)	Gene bank accession number	References
*CKM*	163	Forward	5’TGGGGCTGCAGAAGATTGAG	>XM_012190548.4 (*Ovis aries*)	(67)
		Reverse	3’TCGAACTTGGGATGCTTGCT		
*FGF2*	147	Forward	5’GTGCAAACCGTTACCTTGCT	>NM_001009769.1 (*Ovis aries*)	(4)
		Reverse	3’GTGCCACATACCAACTGGAGTA		
*FLT1*	185	Forward	5’CCGAAGGGAAGAAGGTGGTC	>XM_027973633.3 (*Ovis aries*)	(4)
		Reverse	3’TGTCGTCTCGCAGGTCAAAA		
*GAMT*	183	Forward	5’TCATTCGGGACCATGCCTTC	>XM_015096020.4 (*Ovis aries*)	(12)
		Reverse	3’AGACCTGCGTGCGTATGTTA		
*GATM*	132	Forward	5’AAAGACTACTTCCGCCGTGG	>XM_004010649.5 (*Ovis aries*)	(12)
		Reverse	3’AAACTTTCCCTGAGCAGCCA		
*SLC2A1*	158	Forward	5’CTGTCGTGTCGCTGTTTGTG	>XM_027968628.3 (*Ovis aries*)	(68)
		Reverse	3’AAAGATGGCCACGATGCTCAA		
*SLC6A8*	192	Forward	5’GTGTCTGGAAGGGGGTCAAG	>XM_027962936.2 (*Ovis aries*)	(12)
		Reverse	3’TCTGTGTCCCTGCGTCAATC		
*SLC7A1*	149	Forward	5’AGAACCCGGACATATTCGCC	>XM_060394468.1 (*Ovis aries*)	(69)
		Reverse	3’TTTCACAAACCCCGACACCA		
*ODC1*	127	Forward	5’GTGGTGGCTTTCCTGGATCT	>XM_015094220.4 (*Ovis aries*)	(70)
		Reverse	3’CTGCCTGGCTCAGCTATGA		
*VEGFA*	144	Forward	5’CTTGCCTTGCTGCTCTACCT	>XM_012100430.4 (*Ovis aries*)	(4)
		Reverse	3’GTCCACCAGGGTCTCAATGG		
*RPS18*	174	Forward	5’AGTTCCAGCACATCTTGCGA	>XM_004018745.5 (*Ovis aries*)	(71)
		Reverse	3’GTTCCACCTCGTCCTCAGTG		

**Table 2:** Temperature cycles used in the steps of RT-qPCR reactions.

Stages	Temperature (°C)	Time	Cycles
Holding phase	95 °C	10 min	40 cycles
Denaturation phase	95 °C	15 seg	
Annealing phase	60 °C	1 min	
Extension phase	95 °C	15 seg	
Melting curve phase	60 °C	1 min	
	95 °C	15 seg	

### 2.12 Statistical analysis

Statistical analyses were performed using Statistica Software, version 13.4.0.14 (2018; TIBCO Software, Inc., Palo Alto, CA, United States). The data were initially verified for normality using the Shapiro–Wilk test and for homogeneity of variances using Bartlett’s test. If these conditions were not met, a log10 × transformation was applied. Data from dry matter intake and TMR feed sorting were subjected to analysis of variance (ANOVA) using General Linear Model (GLM) procedures, considering nutritional group (GATMR and BSTMR), week of gestation (Time effect), and their interaction as main effects. The subgroup ‘ewe weight’ was used as a covariate in the model, and results were presented as corrected least square means.

For parameters assessed by ultrasonography, data were analyzed using GLM procedures for repeated-measures of ANOVA, and the effects tested included group, ultrasonography assessment interval, and the interaction between group and assessment interval. The recorded anatomical images (1–3) were considered repeated measures. For placenta and cotyledon data recorded at lambing, the GLM ANOVA effect was nutritional group. For these analyses, ewe weight at lambing was used as a covariate in the model, and means were presented as corrected least squares means. Metabolite markers were analyzed using GLM ANOVA with group, assessment interval (time effect), and the interaction between group and time as effects. Cotyledonary vascular measurements and gene expression were subjected to GLM ANOVA with group as the main effect.

## 3 Results

### 3.1 Feed intake and feed sorting behavior

**Figure 2:**
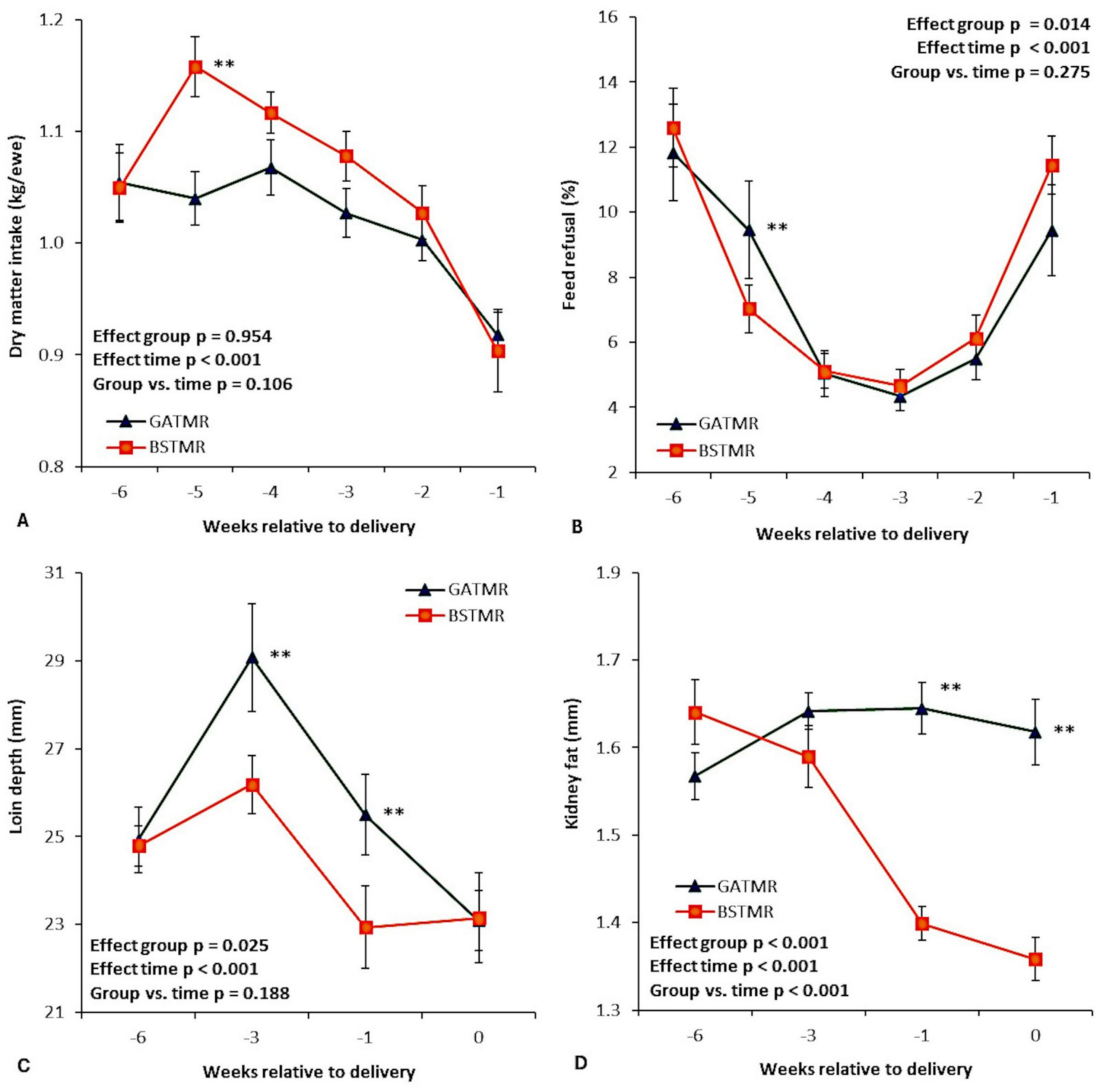
Dry matter intake **(A)** and feed refusal **(B)**. Carcass markers, loin depth **(C)** and kidney fat **(D)**. All parameters were measured during late pregnancy in twin-bearing ewes fed the baseline TMR diet (BSTMR) or TMR diet supplied with rumen-unprotected guanidinoacetic acid (GATMR). Values are represented as means ± SEM. Asterisks indicate where differences between groups occurred (*p < 0.05, **p < 0.01). Time, ANOVA effect for pregnancy weekly intervals.

During the experimental period (Figure 1), changes in feed intake were observed in the animals. Figure 2A shows the dry matter intake from the TMR throughout the experimental interval. In the fifth week before lambing (WBL), the GATMR group recorded lower feed intake than the non-supplemented group (*p* < 0.01), whereas in the subsequent weeks, both treatments showed a reduction in dry matter intake (Time effect, *p* < 0.001). Still in the fifth WBL, the GATMR group recorded higher feed refusal (Figure 2B), although the overall mean was lower than that of the control (6.8 ± 0.4% vs. 8.3 ± 0.4%, *p* = 0.014). This parameter showed a progressive reduction until the fourth WBL and then recorded successive increases in orts until lambing (Time effect, *p* < 0.001).

### 3.2 Carcass marker dynamics

Loin depth (Figure 2C) increased more between the sixth and third WBL in the GATMR group (*p* < 0.01), after which both groups showed a reduction until lambing (Time effect, p < 0.001). The average depth was higher in the GATMR group than in the BSTMR group (25.7 ± 0.5 mm vs. 24.3 ± 0.4 mm, *p* = 0.025). For perirenal fat thickness (Figure 2D), there was an interaction between group and measurement interval due to the reduction observed in the BSTMR group from the third WBL onwards, which did not occur in the supplemented group, resulting in higher values (p < 0.01). Consequently, the overall mean was higher in the GATMR group than in the BSTMR group (1.6 ± 0.02 mm vs. 1.5 ± 0.02 mm, p < 0.001).

### 3.3 Peripheral metabolite effort and GPx oxidative marker

**Figure 3:**
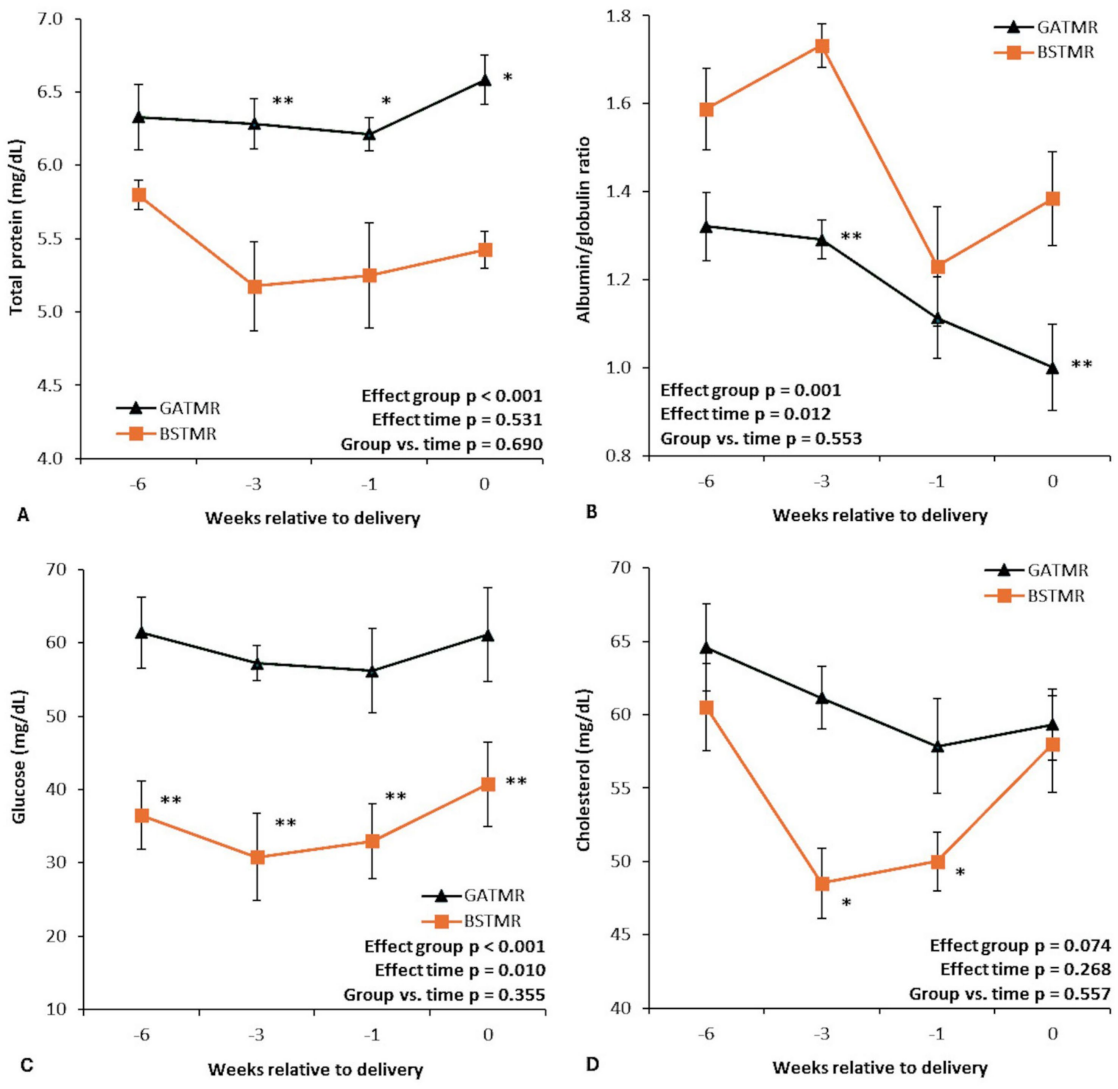
Peripheral levels of total protein **(A)**, albumin/globulin ratio **(B)**, glucose **(C)**, and cholesterol **(D)**, measured during late gestation in twin-bearing ewes fed the baseline TMR diet (BSTMR) or TMR diet supplied with rumen-unprotected guanidinoacetic acid (GATMR). Values are represented as means ± SEM. Asterisks indicate where differences between groups occurred (*p < 0.05, **p < 0.01). Time, ANOVA effect for pregnancy weekly intervals.

Table 3 and Figure 3 summarize the results for these parameters. No differences were observed between groups (Table 3) for plasma levels of triglycerides, urea, creatinine, glutamic-oxaloacetic acid transaminase, glutamic-pyruvic acid transaminase, or glutathione peroxidase. For albumin (Table 3), globulin (Table 3), total protein (Figure 3A), and glucose (Figure 3C), the GATMR group showed higher plasma concentrations than the BSTMR group. For cholesterol in weeks 3 and 1 WBL, the GATMR group also showed higher levels (Figure 3D *p* < 0.05). The albumin/globulin ratio (Figure 3B) showed a reduction over the interval in the GATMR group, with mean values lower than those of the control group (1.2 ± 0.04 vs. 1.5 ± 0.09, *p* = 0.001).

**Table 3:** Peripheral metabolite patterns measured during late gestation in twin-bearing ewes fed the baseline TMR diet (BSTMR) or TMR diet supplied with rumen-unprotected guanidinoacetic acid (GATMR).

Metabolites	Group			*p*-value		
	BSTMR	GATMR	SEM	Group	Time	G x × T
*Energy effort*						
Triglycerides, mg/dL	26.0	20.5	1.537	0.267	0.754	0.662
*Oxidative marker*						
Glutathione peroxidase, U/L	153.4	152.4	0.616	0.651	0.233	0.894
*Proteins*						
Albumin, mg/dL	3.2	3.4	0.062	0.032	0.029	0.643
Globulin, mg/dL	2.2	2.9	0.093	<0.001	0.212	0.737
*Kidney injury*						
Urea, mg/dL	17.8	17.5	0.503	0.760	0.448	0.978
Creatinine, mg/dL	1.3	1.3	0.018	0.907	0.752	0.938
*Liver injury*						
GOT, U/L	74.4	70.7	3.740	0.667	0.320	0.761
GPT, U/L	17.2	16.9	0.586	0.736	0.493	0.478

GOT, glutamic-oxaloacetic acid transaminase; GPT, glutamic-pyruvic acid transaminase; SEM: xxxxx; Time: ANOVA effect for weeks of pregnancy intervals.

Blood samples were collected on days 100, 120, 140, and 145 of gestation.

### 3.4 Uterine and umbilical vascular system dynamics

**Figure 4:**
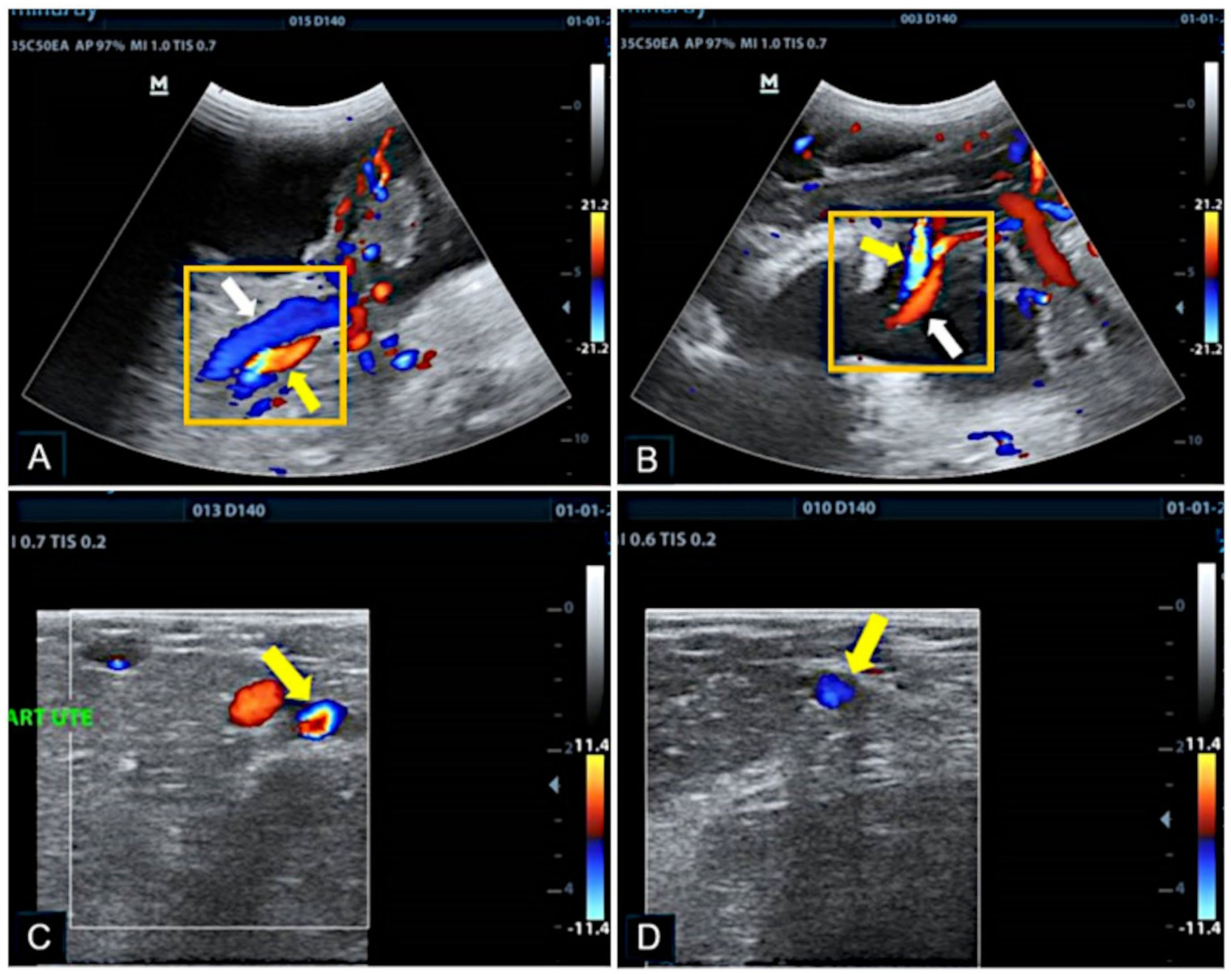
Color Doppler ultrasound image showing the umbilical **(A,B)** artery (yellow arrow), vein (white arrow), and uterine artery **(C,D)** at 140 days of pregnancy, in baseline TMR diet (BSTMR), **(A,C)** and TMR diet supplied with rumen-unprotected guanidinoacetic acid (GATMR) **(B,D)**.

In both groups, the diameter of the uterine artery (Figures 4A,B; Table 4) showed a positive association with gestational age (time effect, *p* < 0.001). Although the GATMR group had a smaller artery diameter, there were no differences between groups for systolic or diastolic peaks (Table 4).

**Table 4:** Uterine artery size and its Doppler hemodynamic parameters, placentome surface area, umbilical vascular system, and Doppler hemodynamic parameters of the umbilical artery measured by ultrasonography during late gestation in twin-bearing ewes fed the baseline TMR diet (BSTMR) or TMR diet supplied with rumen-unprotected guanidinoacetic acid (GATMR).

Attributes	Group			p-value		
	BSTMR	GATMR	SEM	Group	Time	G × T
*Uterine artery attributes*						
Uterine artery diameter, mm	5.9	5.4	0.135	0.034	0.008	0.683
Peak systolic velocity, cm/s	28.6	28.7	0.189	0.919	0.727	0.446
End-diastolic velocity, cm/s	14.4	14.4	0.889	0.981	0.761	0.538
*Umbilical vascular development*						
Artery diameter, mm	6.3	5.8	0.071	<0.001	< 0.001	0.767
Vein diameter, mm	5.2	4.6	0.055	<0.001	< 0.001	0.937
Total vascular area, mm^2^	105.1	86.6	2.060	<0.001	< 0.001	0.923
*Umbilical artery hemodynamic attributes*						
Peak systolic velocity, cm/s	49.4	49.2	0.286	0.708	0.026	0.021
End-diastolic velocity, cm/s	24.6	24.1	0.900	0.937	0.593	0.067
Fetal heart rate, bpm	127.4	116.0	3.003	0.030	0.205	0.734
*Placentome growth features*						
Placentome diameter, mm	17.7	17.3	0.271	0.520	<0.001	0.651
Carunculae thickness, mm	7.5	6.7	0.101	<0.001	0.002	0.537

Time: ANOVA effect for weeks pregnancy intervals.

Measurements were performed on days 100, 110, 120, 130, 140, and 145 of gestation.

The GATMR group exhibited lower values (*p* < 0.001) for the diameter of the umbilical artery and vein (Figures 4A,B), as well as for the total vascular area (Table 4). All parameters increased in both groups over time (Time effect, *p* < 0.001). For the hemodynamic parameters of the umbilical artery (Table 4), no differences were observed in the diastolic peak, whereas the systolic peak showed a group × time interaction due to a reduction in the GATMR group from the second WBL. Fetal heart rate was also lower in animals supplemented with GAA.

### 3.5 Placentome development, reproductive and cotyledonary traits

The placentome diameter (Table 4) decreased in the last two WBL for both groups, as did the caruncle thickness (time effect, *p* < 0.001), with the latter being lower in the supplemented group (*p* < 0.001).

There was no difference between treatments for gestation length, lamb birth weight, placental weight, or placental efficiency (Table 5). For cotyledonary parameters, the GATMR group showed lower total cotyledonary weight (Tables 5, *p* = 0.016), while groups did not differ in total number, cotyledonary area, or cotyledonary efficiency. Table 6 shows the average individual weights, number, and cotyledonary area by morphological type. In this case, GATMR exhibited lower weight and area than BSTMR in subtype B, whereas BSTMR expressed a higher number of cotyledons in subtype A, compared to the control.

**Table 5:** Reproductive traits, lamb weight, and cotyledonary attributes measured at lambing in twin-bearing ewes fed the baseline TMR diet (BSTMR) or TMR diet supplied with rumen-unprotected guanidinoacetic acid (GATMR).

Attributes	Group			*p*-value
	BSTMR	GATMR	SEM	Group
*Reproductive traits*				
Day of pregnancy, days	147.3	147.6	0.553	0.436
Lambs’ weight at delivery, kg	3.2	3.3	0.121	0.627
Placenta weight, g	836.9	668.8	49.32	0.292
Placental efficiency*	3.9	5.1	0.252	0.123
*Cotyledons attributes*				
Total cotyledon weight, g	239.6	228.4	11.53	0.016
No of cotyledons, *n*	77.1	86.0	1.550	0.945
Average cotyledon surface area, mm^2^	481.3	400.6	35.73	0.798
Total cotyledon surface area, mm^2^	36800.3	34676.0	2.877	0.785
Cotyledon efficiency**	91.5	99.3	7.417	0.498

^*^Lamb weight/placental weight ratio; ^**^lamb weight/cotyledons surface area ratio.

**Table 6:** Cotyledon subtype attributes recorded at lambing in twin-bearing ewes fed the baseline TMR diet (BSTMR) or TMR diet supplied with rumen-unprotected guanidinoacetic acid (GATMR).

Attributes	Cotyledon subtype				SEM
	A	B	C	D	
*Cotyledon average weight, g*					
BSTMR	2.3a	3.8bA	4.3b	2.4a	0.109
GATMR	1.9a	2.8bB	3.7c	2.7b	0.114
*Cotyledon number, n*					
BSTMR	22.6aA	21.2a	13.0b	20.3a	0.953
GATMR	29.4aB	22.3b	13.7c	20.5b	1.247
*Cotyledon surface area, mm^2^*					
BSTMR	465.5a	699.5bA	817.8b	511.3a	22.90
GATMR	416.6a	542.9bB	775.3c	536.1b	19.51

a, b, c: Columns with different letters between subtypes for each group, differ (*p* < 0.05); A, B: rows with different letters between groups for each subtype, differ (*p* < 0.05).

### 3.6 Fetal cotyledonary capillary structure

**Figure 5:**
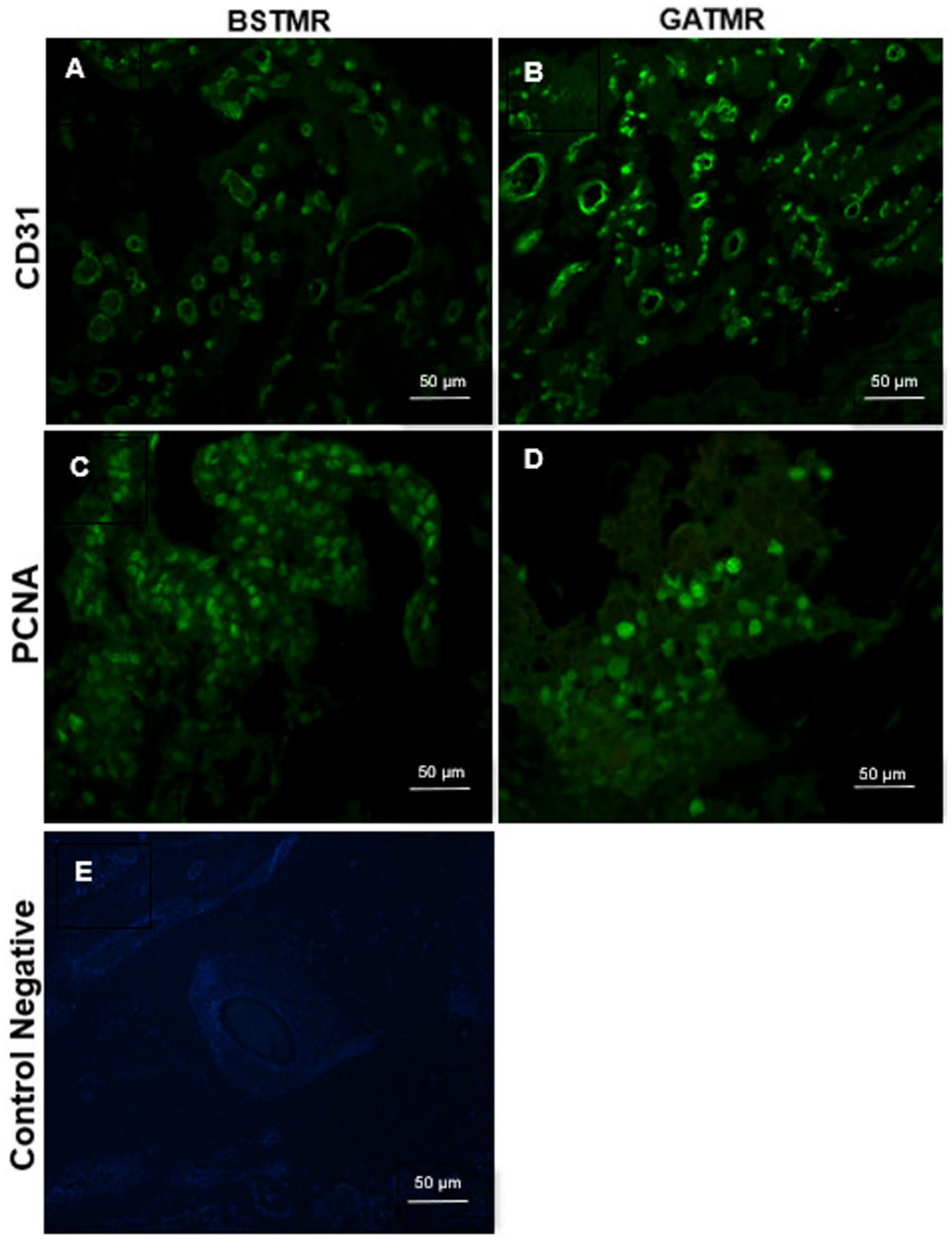
Representative images of immunofluorescence staining in cotyledons for CD31 **(A,B)** and PCNA **(C,D)**. **(A–C)** baseline TMR diet (BSTMR); **(B–D)** TMR diet supplied with rumen-unprotected guanidinoacetic acid (GATMR). **(E)** Negative control. Green fluorescence: CD31 or PCNA-positive cells. Area of image processing: 16.000 μm^2^.

**Figure 6:**
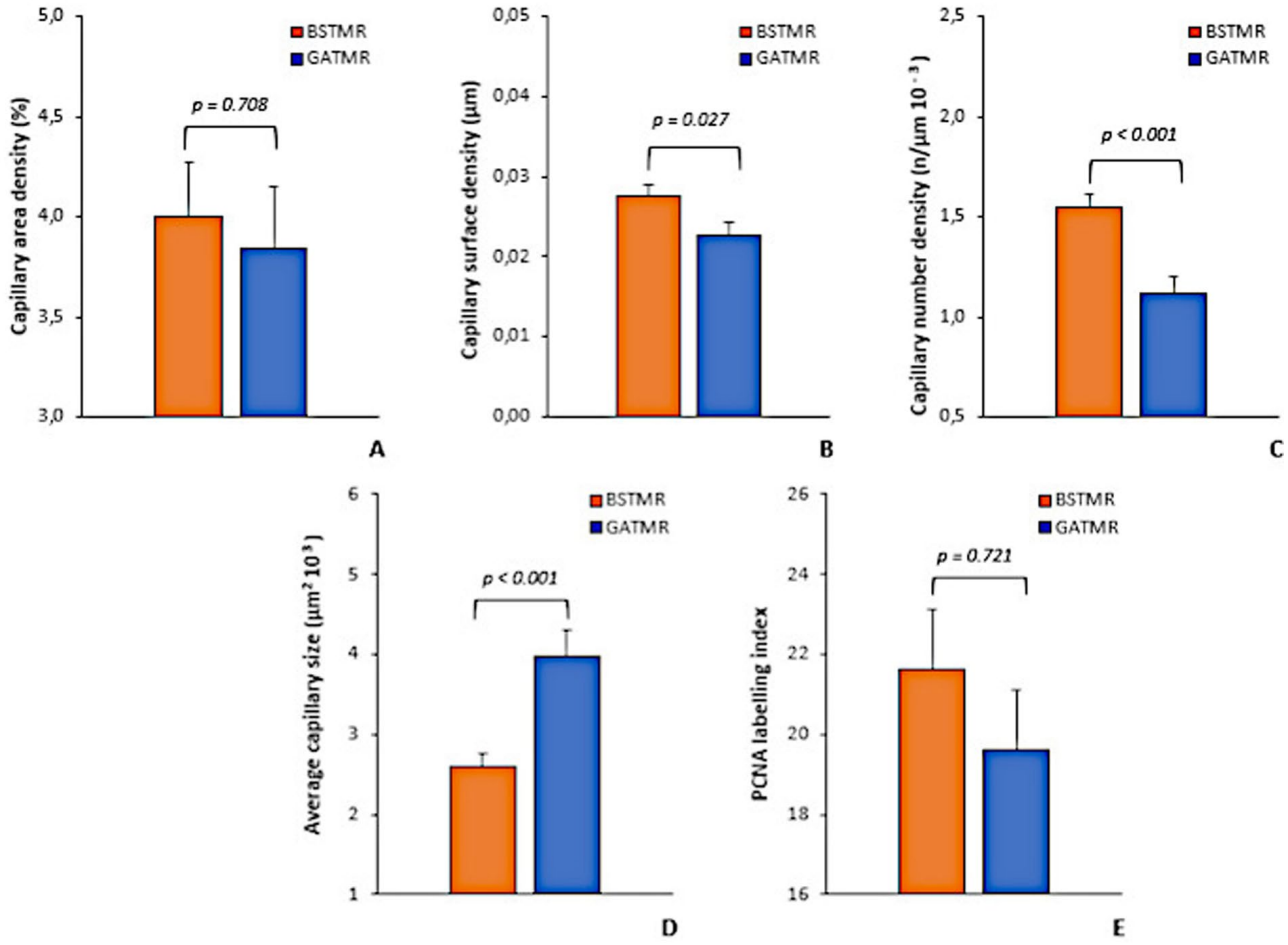
Quantification of cotyledonary vascular measurements by immunofluorescence staining in cotyledons with CD31 and PCNA probes, performed at lambing in twin-bearing ewes fed the baseline TMR diet (BSTMR) or TMR diet supplied with rumen-unprotected guanidinoacetic acid (GATMR). **(A)** capillary area density (%) (total capillary area as a proportion of tissue area); **(B)** capillary surface density in μm (total capillary circumference per tissue area); **(C)** capillary number density (total number of capillaries per tissue area); **(D)**, area per capillary in μm^2^ (average cross-sectional area per capillary); 5E, proportion of proliferating cells by immunohistochemical staining PCNA probe. Values are represented as means ± SEM.

Figure 5 shows images of immunofluorescence staining in cotyledons for CD31 and PCNA probes, and Figure 6 shows the results of cotyledonary capillary system measurements. No differences were observed in capillary area density (CAD, Figure 6A). Animals supplemented with GAA showed reduced capillary surface area density (CSD, Figure 6B) and capillary number density (CND, Figure 6C). The mean capillary size (APC) was higher in the GATMR group (Figure 6D). The cell proliferation rate, measured by PCNA, did not differ between groups (Figure 6E).

### 3.7 Quantitative gene expression for cotyledonary angiogenic markers

**Figure 7:**
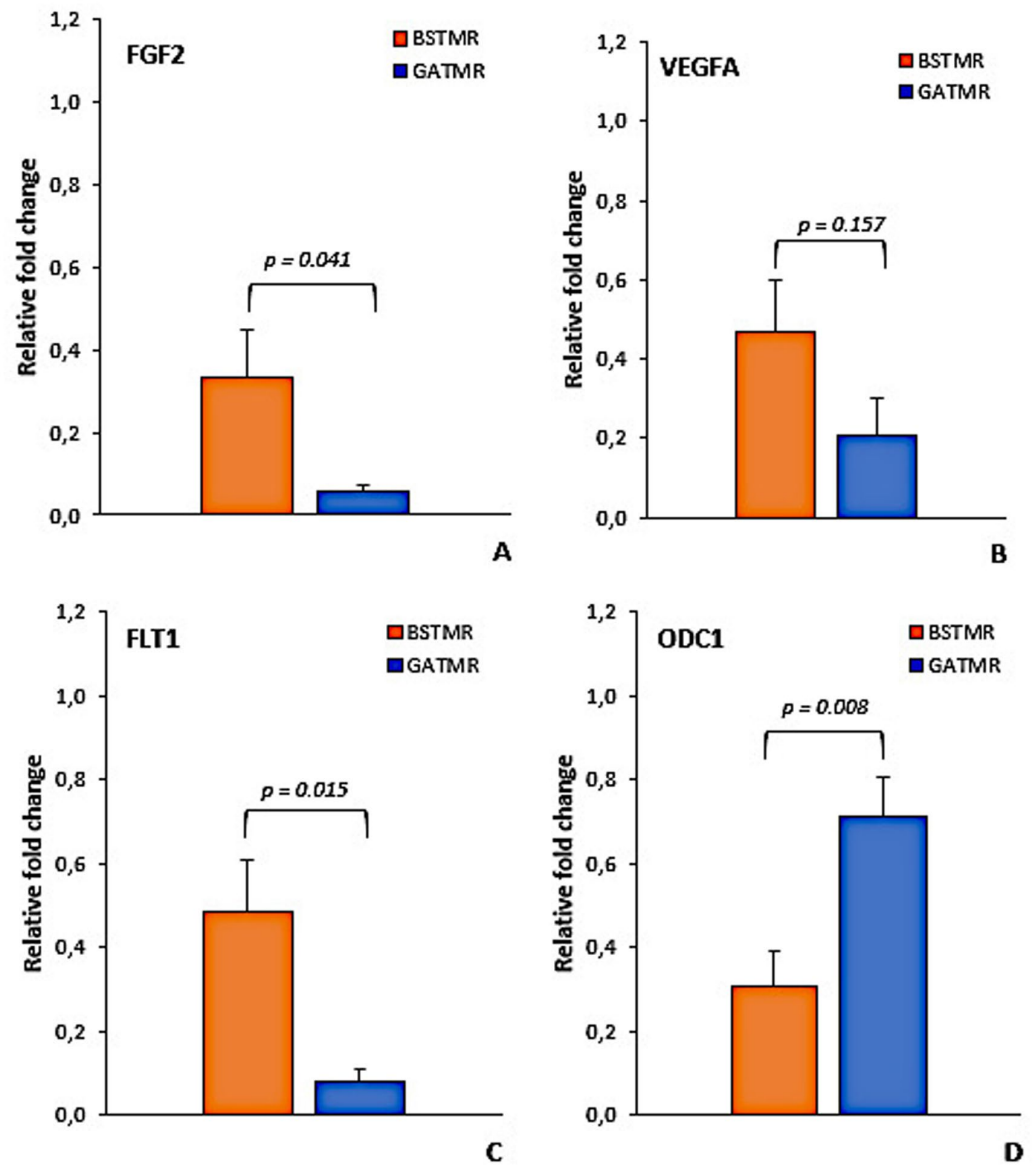
Messenger RNA relative abundance of gene markers involved in angiogenic regulation in cotyledon tissue at lambing in twin-bearing ewes fed the baseline TMR diet (BSTMR) or TMR diet supplied with rumen-unprotected guanidinoacetic acid (GATMR). **(A)** FGF2 (fibroblast growth factor 2); **(B)** VEGFA (Vascular Endothelial Growth Factor); **(C)** FLT1 (Fms related receptor tyrosine kinase 1); **(D)** ODC1 (Ornithine Decarboxylase). Values are represented as means ± SEM; *p*-value indicates differences between groups. Arbitrary units are defined as abundance relative to the mean of RPS18 RNA.

Among the mRNA-encoding genes involved in angiogenesis, two of them, *FGF2* (Figure 7A) and *FLT1* (Figure 7C), were less expressed in the GATMR group. The third gene, *VEGFA* (Figure 7B), also showed lower expression in the GATMR group (*p* = 0.157), although the difference was not statistically significant (*p* > 0.05). For the *ODC1*, synthesis enzyme¸ (Figure 7D), transcript abundance was higher (*p* < 0.01) in the GATMR group than in the BSTMR group.

### 3.8 Cotyledonary expression of nutrient transporter genes and creatine metabolism regulators

**Figure 8:**
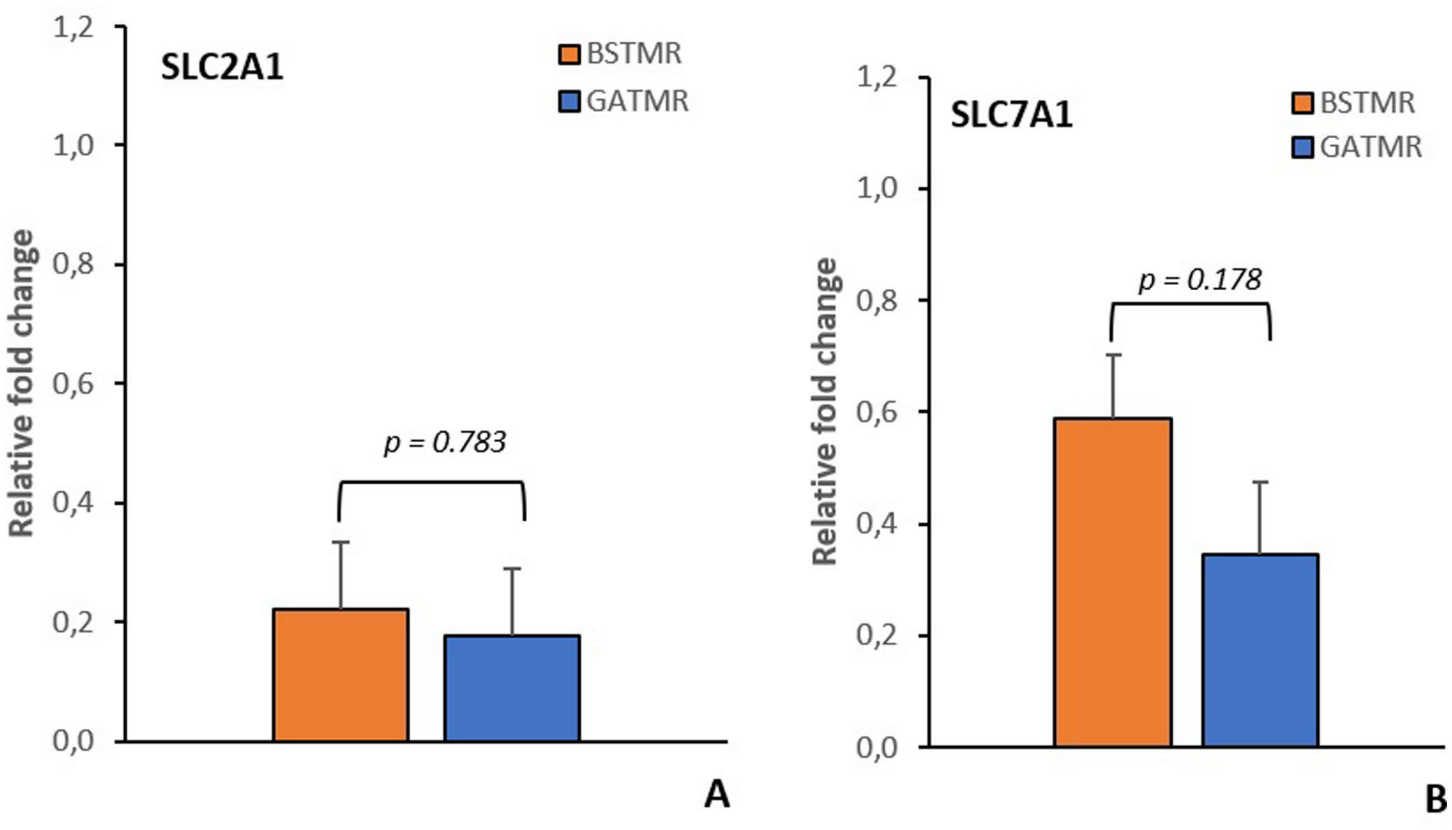
Messenger RNA relative abundance of gene markers involved in glucose and amino acid transporter regulation measured in cotyledon tissue at lambing in twin-bearing ewes fed the baseline TMR diet (BSTMR) or TMR diet supplied with rumen-unprotected guanidinoacetic acid (GATMR). **(A)** SLC2A1 (glucose transporter gene); **(B)** SLC7A1 (amino acid transporter gene). Values are represented as means ± SEM; *p*-value indicates differences between groups. Arbitrary units are defined as abundance relative to the mean of RPS18 RNA.

Regarding the transcripts of the *glucose transporter gene SLC2A1* (Figure 8A) and the *amino acid transporter gene SLC7A1* (Figure 8B), no significant differences were detected between groups. However, both markers showed lower mRNA abundance in the GATMR group.

**Figure 9:**
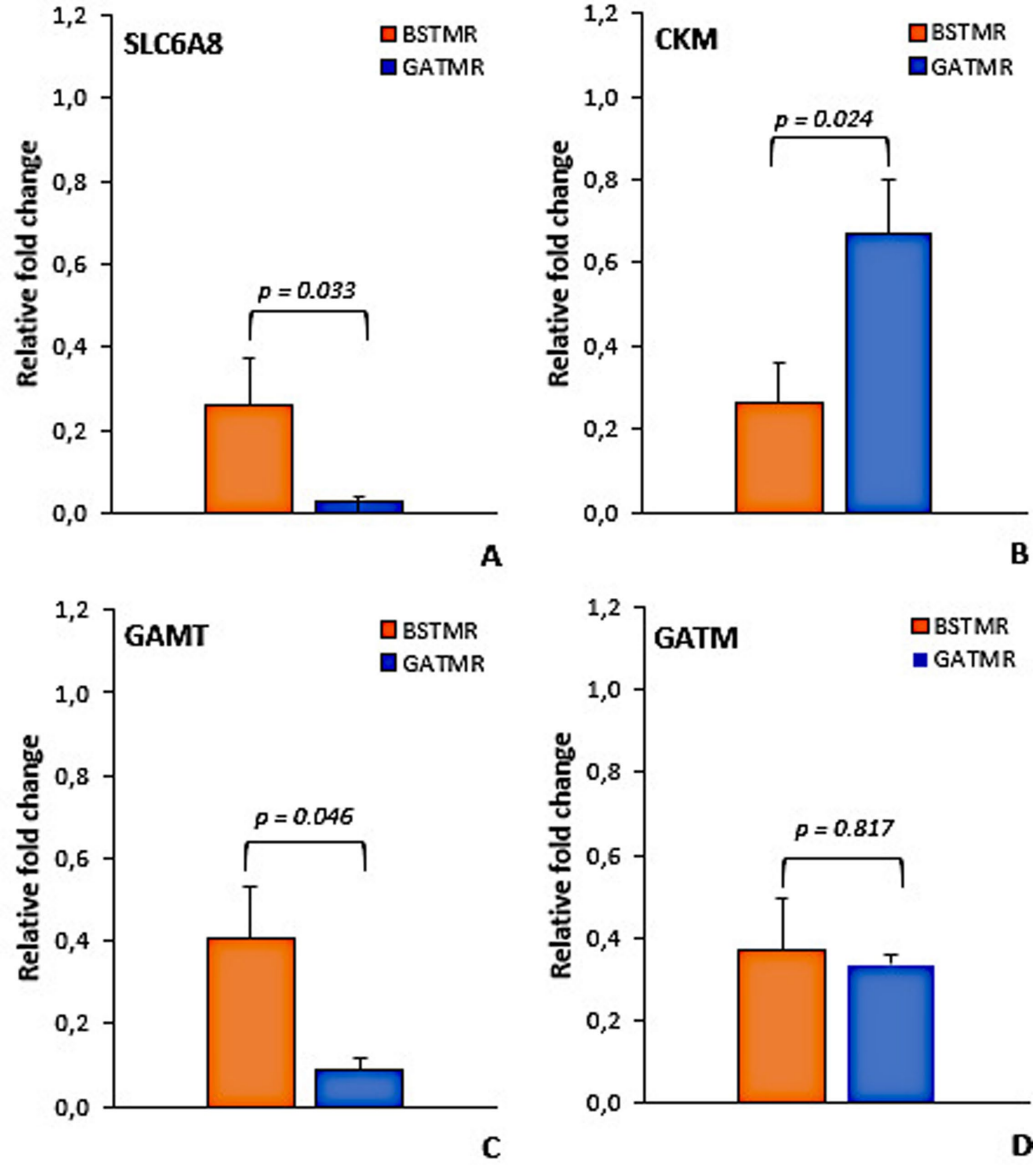
Messenger RNA relative abundance of gene markers involved in creatine transport and synthesis measured in cotyledon tissue at lambing in twin bearing ewes fed the baseline TMR diet (BSTMR) or TMR diet supplied with rumen-unprotected guanidinoacetic acid (GATMR). **(A)** SLC6A8 (Solute Carrier Family 6 Member 8); **(B)** (creatine kinase CKM gene); **(C)** GAMT (Guanidinoacetate N-methyltransferase); **(D)** GATM (glycine amidinotransferase). Values are represented as means ± SEM; p-value indicates differences between groups. Arbitrary units are defined as abundance relative to the mean of RPS18 RNA.

The *creatine transporter marker SLC6A8* (Figure 9A) and the synthesis regulator GAMT (Figure 9C) were lower in the GATMR group, while another synthesis marker, *GATM* (Figure 9D), did not differ between groups. The fetal creatine regulator related to energy release, *CKM* (Figure 9B), was higher (*p* < 0.05) in the GATMR group than in the control.

## 4 Discussion

The results demonstrated that AGA is associated with effects during gestation in ewes carrying twin pregnancies. As a precursor of creatine, GAA appeared to modulate the dynamics of energy reserve depletion, preserving perirenal adipose tissue at the expense of loin muscle mass. This phenomenon was sufficient to support the increases observed in peripheral glucose, protein, and cholesterol levels. Furthermore, the evidence suggested that the metabolic demand generated by the exogenous supply of the creatine precursor was mediated by the animals throughout pregnancy via particle selectivity during feeding, as an adaptive response to the newly established homeostatic balance.

As a nutritional additive, GAA has been widely studied for its effects on feed conversion efficiency and carcass performance in animal production systems. In poultry, it improves weight gain and carcass yield (26, 27), and in swine, improves meat quality and reproductive performance (28, 29).

At the end of gestation, the sequential occurrence of multiple events, such as preparation for lambing and milk production, requires metabolic adjustments that mobilize body reserves to supply energy and amino acids. Adipose stores decrease due to increased lipolysis and triacylglycerol mobilization, and reduced lipoprotein lipase activity, thereby favoring energy allocation to the fetus and lactation. These processes correlate with elevated placental hormone levels and the consequent development of insulin resistance (30). As a result, the pancreas promotes compensatory hyperinsulinemia, inducing metabolic effects such as visceral fat accumulation (31). In this context, increased pancreatic insulin secretion during the catabolic phase may have helped preserve renal fat. Although the correlation between GAA effects and glycemic indices remains unclear, there are indications that the creatine precursor may exert an insulinotropic effect by sharing transporters with beta-guanidinopropionic acid, an anti-hyperglycemic therapeutic agent. However, the energetic cost of creatine synthesis may also stimulate compensatory hepatic glucose production (25). Cholesterol alterations may be associated with excess homocysteine, as described in humans (32).

In addition, animals receiving GAA may have reduced their reliance on fat mobilization as a primary energy source due to increased protein indices. We also observed that loin depth was greater between the 6th and 3rd WBL in ewes receiving GAA, then decreased. In cows, reductions in protein mobilization, specifically in the ribeye area, have also been reported (24), since GAA supplementation spares arginine, which regulates the mechanistic target of rapamycin (mTOR) signaling pathway and prevents protein degradation. Metabolic indices reflect the nutritional status of the organism; in this study, total protein values suggest that exogenous GAA spared amino acids that would otherwise be directed toward protein synthesis (33). Additionally, we hypothesize that increased lumber depth may reflect a greater presence of intramuscular fat, which is not distinguishable on ultrasonography. In this regard, Jin et al. (28) described higher expression of acetyl-acetyl coenzyme A (CoA) in the *longissimus dorsi* muscle of sheep supplemented with GAA, suggesting that GAA may have stimulated fatty acid synthesis in muscle tissue by increasing acetyl-CoA and contributing to intramuscular fat development.

The consistent metabolic effort recorded in this study appeared well tolerated, with serum urea and creatinine concentrations remaining within the expected range for ewes with twin pregnancies (29). Hepatic and renal markers did not differ; GOT and GPT plasmatic levels were similar between groups and remained within the reference range for twin-bearing ewes. Inflammatory indicators, such as the albumin/globulin ratio, decreased and approached ideal values throughout gestation. Glutathione peroxidase did not show significant fluctuations and remained similar between groups (34). Multiple gestation in sheep is a pregnancy model with limited knowledge of nutritional requirements, making it particularly challenging to optimize feeding strategies. Insufficient energy intake in late gestation induces negative energy balance and hypoglycemia, which can lead to complications such as pregnancy toxemia (35) due to rapid lipid mobilization needed to sustain the final growth of the fetuses (36, 37). These animals typically show high sensitivity to external nutritional stimuli, such as novel metabolic modulators like GAA, which require careful modeling to address the multiple events occurring at the end of gestation, thereby ensuring fetal development and maternal metabolic adaptation for lambing and lactation. More specifically, the ruminant placenta is considered an ideal model for studying placental development because the maternal and fetal portions remain closely associated but structurally independent throughout gestation, allowing each tissue to be evaluated separately (38).

At the end of gestation, glucose turnover increases due to greater efficiency of hepatic gluconeogenesis, and in ewes with multiple pregnancies, gluconeogenesis appears to compensate for the increased energy demand associated with fetal development (39). Our main objective was to investigate the potential of GAA as support for maternal energy demand at the end of gestation in ewes with twin pregnancies, based on vascular communication between the mother and the fetus, since nutritional transport plays a fundamental role in the formation and development of the vascular system necessary to sustain the high nutritional needs of the fetus in the final growth phase (40). Collectively, these nutritional interventions aim to maximize the flow and efficiency of amino acid use for protein synthesis. Indeed, creatine plays an important role in energy metabolism and protein synthesis, storing energy as creatine phosphate in tissues such as skeletal muscle, the brain, and the reproductive tract (16), and GAA has recently been used as a source for endogenous creatine synthesis and as a strategy to spare amino acids, such as arginine, for other functions in organs with high metabolic activity (24). Arginine has direct effects as a source of amino acids for placental growth and for regulating mTOR activation to inhibit protein degradation (41, 42), in addition to acting as a precursor for the synthesis of nitric oxide (NO), a vasodilator and angiogenic factor associated with increased placental blood flow (43), while methionine acts as a metabolic regulator for placental growth and fetal support (44). The use of GAA in the diet has been shown to promote mTOR/Akt signaling activation and increase protein synthesis in cattle (45) and sheep (46), but the hemodynamic effects influencing gestation performance and fetal growth have been poorly described.

In this sense, the evidence suggests that the metabolic redirection induced by GAA supplementation served as a signaling trigger in the vascular system at the placental level, particularly for fetal cotyledonary angiogenesis. In our study, the reduction in uterine artery diameter was not accompanied by changes in its hemodynamic parameters, but it was associated with reduced umbilical and maternal caruncular vascular development, a lower peak systolic volume of the umbilical artery, and, above all, a reduction in cotyledonary surface area and its capillary vascular system. This evidence was supported by underexpression of cotyledonary angiogenic markers *(FGF2, FLT1, VEGFA)* and a stronger expression of the antiangiogenic factor *ODC1*. These results may indicate lower vascular impedance and, consequently, greater arterial blood flow (47), given the negative associations between Doppler indices and blood flow to downstream organs (48). In sheep, umbilical blood flow increases throughout gestation, accompanying fetal growth during the second half of gestation (49), when both the absolute rates and, importantly, the proportions of total uterine and umbilical blood flows received by the caruncular and cotyledonary tissues, respectively, increase as gestation progresses (50).

The placenta is a metabolically active organ whose hemodynamics are regulated according to the availability and demand for nutrients, as an adaptive response that preserves nutrient supply to the fetus (51). Fetoplacental growth depends on adequate and continuous uteroplacental perfusion (52). As gestation progresses, mitochondrial ROS production increases due to increased energy demand and ATP synthesis, and changes in the maternal lipid profile resulting from continuous placental progesterone synthesis exacerbate the cascade of oxidative stress and lipid peroxidation (53). The pro-inflammatory state and oxidative imbalance in late gestation increase superoxide anion (SO^−^) levels, which react with NO, and reducing its bioavailability, impairing endothelial function and vasodilation, and weakening vascular integrity (54). In cows that received GAA, an increase in serum NO and, consequently, an increase in blood flow to the placenta–fetal interface at the end of gestation were observed (19). As for cotyledonary subtypes, our study did not show signs of structural remodeling, since in ovine pregnancies subtypes A and B predominate throughout gestation. In contrast, subtypes C and D are heavier and fewer in number (55), as observed in both groups. It is known that this distribution of placentome subtypes and sizes can be influenced by placental blood flow and oxygen nutritional status, as exposure to maternal undernutrition or hypoxemia in early pregnancy leads to a change from subtypes A to subtypes D in late pregnancy (56, 57), therefore the presence of an increased number of subtypes C and D appears to be a placental adaptation to adverse intrauterine conditions aimed at increasing nutrient supply (24), which was not observed.

Among the signaling pathways important for angiogenesis, *VEGFA* is the most important, as it binds to its positive receptor *KDR* or negative receptor *FLT1*, promoting cell proliferation, differentiation, permeability, vascular tone, and stimulating the production of other vasoactive molecules, such as NO. At the same time, *FGF2* participates in the initial stimulation of placental vascularization (4–6). *VEGFA* is highly expressed by cytotrophoblasts in the first weeks of gestation, whereas hemangiogenic cell cords show the strongest *KDR* immunoreactivity. Deletion of the *VEGFA* or *KDR* gene in mice impairs the initiation of placental vasculogenesis (58, 59), since *VEGFA* is a key endothelial mitogen and its alterations can favor angiogenic remodeling of vessels by stimulating the formation of a capillary network in the mesenchymal core of the villi (59). Alterations in placental growth and vascular development have been associated with changes in the expression of key angiogenic factors, including *VEGF*, as well as endothelial nitric oxide synthase (eNOS), which produces NO (45, 56), as observed in human placental explants with pre-eclampsia, which exhibited increased production of the *VEGF-1* receptor that binds to and inhibits *VEGFA* ligands (60). Along with these regulatory pathways, *IGF-2* participates in placental growth control, and in rodents, its expression is related to reduced placental size under conditions such as nutrient restriction and glucocorticoid administration (8, 9). The *IGF2-H19* gene complex integrates placental nutrient supply with fetal nutrient demands for growth (7) and coordinates the molecular mechanisms by which environmental signals alter placental nutrient transport capacity. Meanwhile, *ODC1*, which was reduced in abundance by GAA, is an enzyme involved in polyamine regulation and, together with mTOR pathways, finely regulates cell growth and autophagy in response to nutrient availability and metabolic demands (61).

An analysis of genetic markers related to creatine availability and nutrient transporters revealed that, despite differences in expression, nutrient flow from mother to fetus may not have been affected by alterations in the cotyledonary vascular system. Moreover, the lower expression of the *SLC6A8* and GAMT markers and the higher expression of *CKM* in the GAA group may indicate a reduction in the creatine synthesis pathway and a more efficient energy conversion at the cellular level via the creatine phosphate system at the end of gestation, since creatine synthesis from GAA occurs through a reaction catalyzed by GAMT, resulting in the production of creatine and S-adenosylhomocysteine. Only then is creatine released into circulation, where it can be absorbed by various tissues (61).

The vascular dynamic changes observed in the GAA-supplemented group, characterized by reductions in uterine and umbilical vascular diameters, total vascular area of cotyledonary capillaries, and umbilical artery systolic peak velocity, occurred without evidence of impaired fetal development. These effects may be associated with increased circulating homocysteine concentrations (Ostojic, 2021). The metabolically challenging condition characteristic of twin-bearing ewes (1) may increase the metabolic demand for folate and vitamin B12 (Cbl), thereby favoring alterations in the homocysteine–folate–Cbl metabolic pathway, which is closely linked to the maintenance of uteroplacental vascular function (62). Although the effect of GAA on increasing circulating homocysteine concentrations has not yet been described in pregnant ruminants, studies conducted in young women demonstrated that supplementation with 2.4 g of GAA per day for 6 weeks significantly increased serum homocysteine concentrations (63).

Regarding fetal heart rate, fetuses from GAA-supplemented ewes showed an approximate 9% reduction, while remaining within the physiological range considered normal for ovine fetuses (64). Studies in ruminants have associated reduced fetal heart rate with fetal hypoxia (65); however, this hypothesis could not be confirmed in the present study since fetal blood gas concentrations were not evaluated. In addition, birth weights were similar between groups (Table 5), suggesting no significant impairment in fetal development.

Dietary supplementation with GAA in ewes carrying multiple pregnancies induces a reprogramming of maternal–fetal physiology, as evidenced by the results. GAA is not limited to of its role as a growth promoter; it is a potent metabolic modulator that prioritizes cellular energy efficiency over placental vascular expansion. The observation of a placenta with lower numerical density (CND) and surface area (CSD) of capillaries, but with larger caliber capillaries (APC), demonstrates an adaptive vascular remodeling process. This fact, where efficiency replaces expansion, is a direct consequence of an alteration in molecular signaling. Our gene expression data reveal an antiangiogenic molecular environment, with reduced expression of the *FGF2* and *FLT1* genes in cotyledonary tissue, indicating a strong indication of attenuated emergence of new vessels. The coordination of the suppression of these pathways constitutes a fundamental mechanism for inducing vascular quiescence and favoring the maturation of the already formed vascular network (66). This angiogenesis regulation process is highly complex, being controlled by a vast network of factors, among which non-coding RNAs stand out, capable of modulating the expression of key genes such as *VEGF* and *FGF*, directly impacting vascular development and stability (67).

This molecular inhibition mechanism is intensified by overexpression of the *ODC1 (Ornithine Decarboxylase)* gene. Polyamines, products of *ODC1*, are recognized as regulators of nitric oxide synthesis in endothelial cells, a key mediator of vascular tone and endothelial function (68). Therefore, increased *ODC1* can influence vascular remodeling not only by modulating proliferation, but also by altering local hemodynamic function. Endothelial function, in turn, is crucial for regulating blood flow and vascular tone, adapting perfusion to tissue needs (69).

In the present study, no evidence was observed that dexamethasone-induced parturition influenced the expression of glucocorticoid-sensitive genes, such as *FGF2*, *FLT1*, and *ODC1*. However, the potential interference of this protocol cannot be ruled out and should be considered a limitation of the study. Therefore, the future investigations are encouraged to avoid the use of pharmacological agents that act directly on uteroplacental function to minimize potential confounding effects. In contrast, the use of only a single endogenous control gene (*RPS18)*, as adopted in the present study, should be considered a limitation. Therefore, the future investigations are encouraged to employ multiple endogenous control genes to provide greater robustness to gene expression analyses.

It is important to highlight that the primary etiology of this vascular reorganization is not intrinsic to the placenta, but rather in the fetal metabolic state. Supplementation with GAA promotes optimization of fetal energy metabolism. It is observed that the underexpression of the creatine transporter *SLC6A8* and the enzyme responsible for its synthesis, *GAMT*, constitutes a negative feedback response, which corroborates the effective absorption and utilization of GAA (70). However, a central aspect of *CKM* overexpression is its superior capacity for energy buffering, which gives fetal cells greater metabolic adaptability. This scenario of high fetal bioenergetic efficiency ensures that the fetus’s vital functions and growth are maintained even with a lower demand for energy substrates. However, it is important to emphasize that the maternal vascular architecture, responsible for placental support, remains a fundamental element of success of the pregnancy, as it ensures the adequate supply of fetal development (71).

## 5 Conclusion

Based on the results, we conclude that, under the experimental conditions of this study, dietary supplementation with 0.9 g/kg of dry matter of GAA in the diet of ewes with twin pregnancies efficiently directs maternal metabolic effort, modifying feeding behavior and prioritizing the maintenance of the animal’s energy reserves. At the same time, the dosage used also affected the development of the maternal–fetal vascular communication system. This latter result suggests the need to refine GAA dosing to better manage this product and meet the demands of multiple pregnancies characterized by metabolic sensitivity and diverse energy requirements.

## Data Availability

The original contributions presented in this study are included in the article. Further inquiries can be directed to the corresponding author(s).
